# Stiffness based enrichment of leukemia cells using microfluidics

**DOI:** 10.1063/1.5143436

**Published:** 2020-07-01

**Authors:** Muhymin Islam, Abhishek Raj, Brynn McFarland, Hannah Maxine Brink, Jordan Ciciliano, Meredith Fay, David Richard Myers, Christopher Flowers, Edmund K. Waller, Wilbur Lam, Alexander Alexeev, Todd Sulchek

**Affiliations:** 1 George W. Woodruff School of Mechanical Engineering, Georgia Institute of Technology, 801 Ferst Drive, Atlanta, Georgia 30332-0405, USA; 2 The School of Biological Sciences, Georgia Institute of Technology, 310 Ferst Drive, Atlanta, Georgia 30332-0535, USA; 3 Wallace H. Coulter Department of Biomedical Engineering, Georgia Institute of Technology, 313 Ferst Drive, Atlanta, Georgia 30332-0535, USA; 4 Winship Cancer Institute, Emory School of Medicine, 1365 Clifton NE Rd.: Atlanta, Georgia 30322, USA

## Abstract

To improve the survival rate of cancer patients, new diagnosis strategies are necessary to detect lower levels of cancer cells before and after treatment regimens. The scarcity of diseased cells, particularly in residual disease after treatment, demands highly sensitive detection approaches or the ability to enrich the diseased cells in relation to normal cells. We report a label-free microfluidic approach to enrich leukemia cells from healthy cells using inherent differences in cell biophysical properties. The microfluidic device consists of a channel with an array of diagonal ridges that recurrently compress and translate flowing cells in proportion to cell stiffness. Using devices optimized for acute T cell leukemia model Jurkat, the stiffer white blood cells were translated orthogonally to the channel length, while softer leukemia cells followed hydrodynamic flow. The device enriched Jurkat leukemia cells from white blood cells with an enrichment factor of over 760. The sensitivity, specificity, and accuracy of the device were found to be >0.8. The values of sensitivity and specificity could be adjusted by selecting one or multiple outlets for analysis. We demonstrate that low levels of Jurkat leukemia cells (1 in $10^{4}$ white blood cells) could be more quickly detected using flow cytometry by using the stiffness sorting pre-enrichment. In a second mode of operation, the device was implemented to sort resistive leukemia cells from both drug-sensitive leukemia cells and normal white blood cells. Therefore, microfluidic biomechanical sorting can be a useful tool to enrich leukemia cells that may improve downstream analyses.

## INTRODUCTION

The detection of cancer cells after a regimen of treatment is an important prognostic factor for disease relapse in patients with *Acute Lymphoblastic Leukemia (ALL)*,^1–3^ *Chronic Lymphocytic Leukemia (CLL)*,^4,5^ and lymphoma.^6^ The ability to detect scarce cancer cells at even lower detection limits, that is, “minimal residual disease” (MRD), would improve the accuracy and confidence of diagnosis.^7,8^ Additionally, simplifying the overall complexity and expense of diagnosis approaches would translate into meaningful improvements in health outcomes. Clinical and research leukemia diagnosis techniques, such as polymerase chain reaction (PCR),^7^ flow cytometry-based analyses,^9^ immunophenotyping and microscopic examination,^10,11^ nanocarriers,^12^ and adhesion-based techniques,^13^ are all limited by the rarity of diseased cells. Deep sequencing-based techniques require highly sophisticated sequencer systems and skilled officials to clinically interpret the data, which are often expensive^14–16^ with a significant time delay of 1–14 days.^16^ Sorting and enriching of rare cancer cells using inertial microfluidics have been reported in the literature.^17,18^ Yet, obtaining high enrichment using biophysical properties of cells is often challenging due to a size overlap with the healthy counterparts.^18^ Thus, a label-free approach for rapid enrichment of low levels of leukemia cells is needed.

The mechanical stiffness of individual human cells can be a key parameter that reveals dysfunction of the cell.^19,20^ The biophysical analysis of leukemia cells showed leukemia and normal blood cells translated through narrow microchannels with different transit times due to differences in deformability.^21^ The inherent biophysical differences of cell types have been effectively exploited previously to isolate and detect numerous malignant cells in microfluidic platforms.^22–29^

**FIG. 1.**
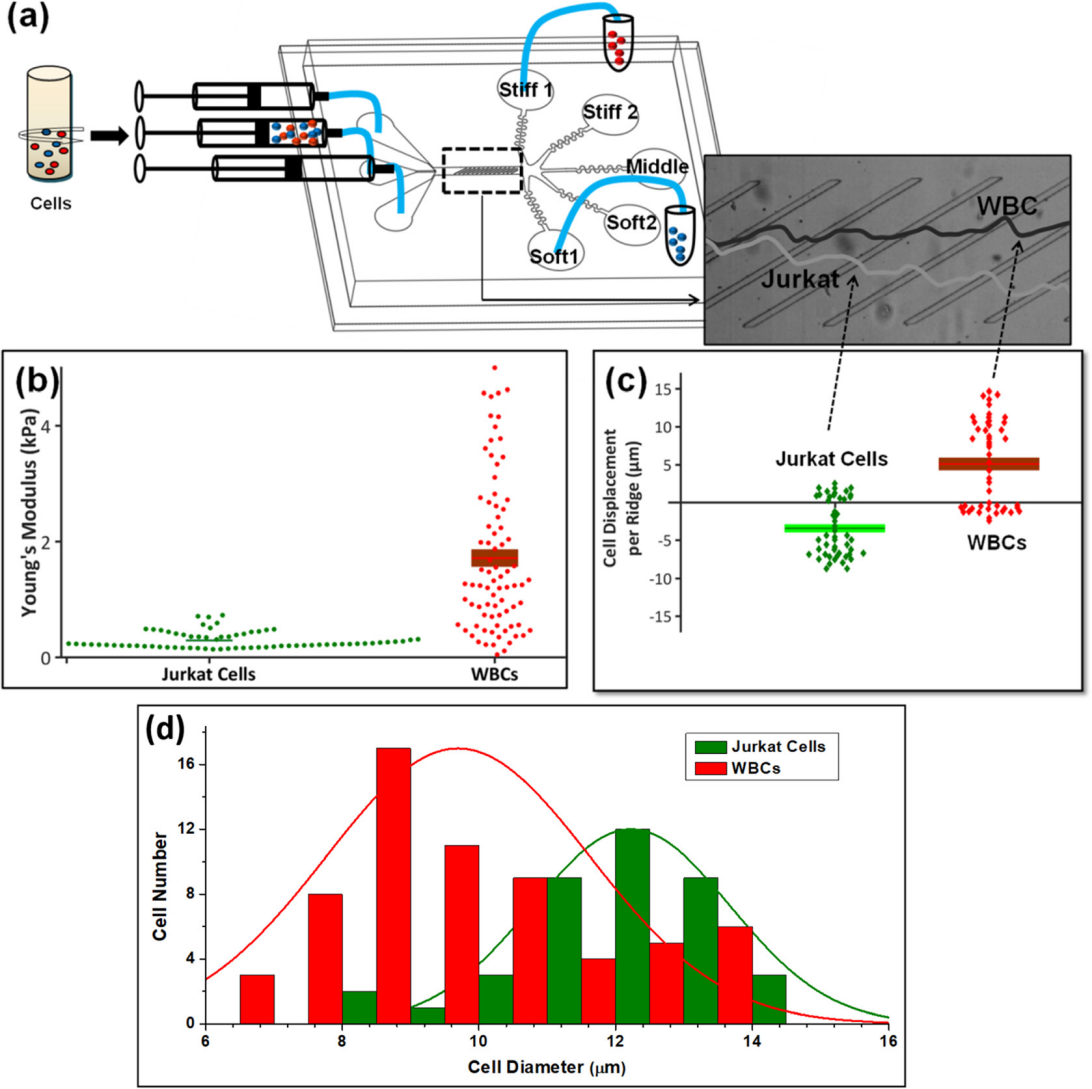
(a) Schematic diagram of the cell sorting device. (b) Young's modulus of Jurkat (green colored) and white blood cells (red colored) measured before infusing into the microfluidic device (p-value < 10^−10^, N = 80), and (c) displacement of Jurkat cells and WBCs run through the device separately (p-value < 1.54 × 10^−10^, N = 50). The representative trajectories of WBC and Jurkat cells moving in the opposite direction are shown in the inset. (d) Cell size distribution for Jurkat cells (shown in green) and WBCs (shown in red colored) shows a significant overlap.

Recently, we have developed microfluidic sorting technology that uses a combination of hydrodynamic and compressive forces to separate and sort individual cells by biophysical properties that include stiffness, size, adhesion, and *viscoelasticity*,^22,30,31^ as well as functional states such as viability^32^ and drug-resistant and drug-sensitive leukemia cells.^33^ The technology consists of a microchannel with periodical, diagonal ridge constrictions [Fig. 1(a)] that deform cells as they flow to modify their trajectory in proportion to cell stiffness. When a soft cell infused into the device encounters the ridge, it either deflects perpendicular to the ridge or moves undeflected. In contrast, a stiff cell deflects along the ridge. This gives rise to distinct trajectories for cells with distinct stiffnesses, and they end up at different outlets. The device is designed to have five different outlets as shown in Fig. 1(a). Soft-1 and soft-2 outlets collect softer cells, whereas stiff-1 and stiff-2 outlets collect stiff cells. The middle outlet receives a mixture of softer and stiffer cells with stiffness values overlapping.

In this study, we demonstrate the utility of the biophysical enrichment technique to improve the sensitivity and speed of detection of a low level of leukemia Jurkat cells [1 Jurkat cell in 10^4^ white blood cells (WBCs)]. White blood cells collected from healthy donors were spiked with various numbers of Jurkat leukemia cells and sorted by ridges, which direct stiff cells along the ridges, while softer cells follow fluid flow streams. The higher stiffness WBCs were translated along the ridges and toward one side of the channel, while softer Jurkat leukemia cells migrate toward the bottom part of the channel driven primarily by hydrodynamic forces [Figs. 1(b) and 1(c)]. We note the cell size for both types substantially overlapped as shown in Fig. 1(d). However, in our previous work,^23^ we have reported that the natural variation in the cell size (for Jurkat cells) has a weak effect on the separation. Following is the reason for the same. The construction of the device is such that cells traversing through the ridge experience an elastic force. This elastic force arises due to the cell deformation.^23^ The cell deformation is more sensitive toward the stiffness of the cells in comparison to their size.^20,23^ Thus, separation efficiency is more sensitive to the stiffness and weakly sensitive to the cell size. Hence, sorting is primarily based on stiffness. Outlets integrated at the channel exits continuously collect the separated cells and, thereby, fractionate cells by biomechanical properties.^32,34^ This approach substantially enriched the spiked cancer cells within a majority population of normal WBCs, with an enrichment of over 760-fold possible and with an accuracy of sorting greater than 0.8. Thus, even for rare cancer cells diluted to a ratio of 1:10 000, downstream detection of these cells was possible.

We have shown in our earlier studies that different leukemia cell models, including Jurkat, K562, and HL-60, differ biophysically from healthy WBCs.^22^ In this study, Jurkat leukemia model cells were mixed with healthy WBCs at different ratios to determine how biophysical enrichment can be used to isolate target leukemia cells. Enrichment was maximized by using five sorting outlets to increase fractionation. A ridge spacing of 200 *μ*m allowed sufficient cell relaxation, and we limited the number of ridges to 14 to avoid compression-induced plasticity. In addition, the sorting process was applied after a treatment of chemotherapy to isolate drug-resistive leukemia cells from drug-sensitive leukemia cells within a majority population of healthy WBCs. The label-free sorting method demonstrates how highly fractionated subpopulations of heterogeneous cells can be used to detect target cell populations and may provide the opportunity to study MRD in patients with acute leukemia to more accurately measure the initial treatment response and to detect relapse earlier.^8^

## RESULTS AND DISCUSSION

### Characterization of Jurkat cells and WBCs

AFM analysis was conducted on both Jurkat cells and WBCs. The average Young's modulus of Jurkat cells was 99 ± 47 Pa, and that of WBCs was 1990 ± 1840 Pa (*p*-value < 10^−10^, *N* = 80), shown in Fig. 1(b). The stiffness of a cell is related to the intrinsic properties of the cell membrane, nucleus, and components of the cytoskeleton.^37–39^ The cells were also processed by the microfluidics, and the trajectories of cells were evaluated by video microscopy. The relative displacement of Jurkat cells and WBCs from a ridge in the channel was −3.4 ± 3.6 *μ*m and 5.1 ± 5.8 *μ*m, respectively. The opposite polarity indicates that the cell types displaced in opposite directions, as shown in Fig. 1(c).

The mechanism of cell separation has been investigated previously as a balance between hydrodynamic drag force and elastic force due to cell compression.^23^ Since Jurkat cells and WBCs have different stiffness values, the cells, thus, experience distinct elastic forces as they pass through ridges, but similar hydrodynamic forces. Softer Jurkat cells experienced a weak elastic force and are directed to the negative transverse direction due to ridge-generated circulatory flow in the microchannel, which causes the fluid near the bottom surface of the channel to move in the negative transverse direction.^34,40^ On the other hand, stiffer WBCs were translated by a strong elastic force along diagonal ridges. Consequently, Jurkat cells and WBCs migrate to opposite sides of the ridged microchannel and separated according to their mechanical stiffness.^23^

Although there is an overlap between Young's modulus of Jurkat cells and WBCs, the cell stiffness differences in majority populations lead to different trajectories and separation to different outlets. We characterized the cell size difference [shown in Fig. 1(d)], provide that for comparison, and find that there is a significant overlap between the cell sizes too. There may be differences in other cell mechanical properties such as viscosity,^22^ which affects separation, although that was not explored in this study. Viscosity of cells does play a role in defining the trajectory depending upon the spacing between two consecutive ridges. Highly viscous cells do not regain their shape after the first few compressions and, hence, follow the path of the flow streamline and end up in the softer outlets, whereas a weakly viscous cell regain its shape during its movement through the spacing between one ridge and another. This allows them to traverse continuously along the ridges, and they are separated to the stiff outlets. Thus, separation efficiency is affected by the differences in the viscosity of cells. This principle has been utilized in our previous work to sort leukemia cell lines K562 and HL60 from a mixture.^22^

### Sorting of leukemia cells from WBCs

**FIG. 2.**
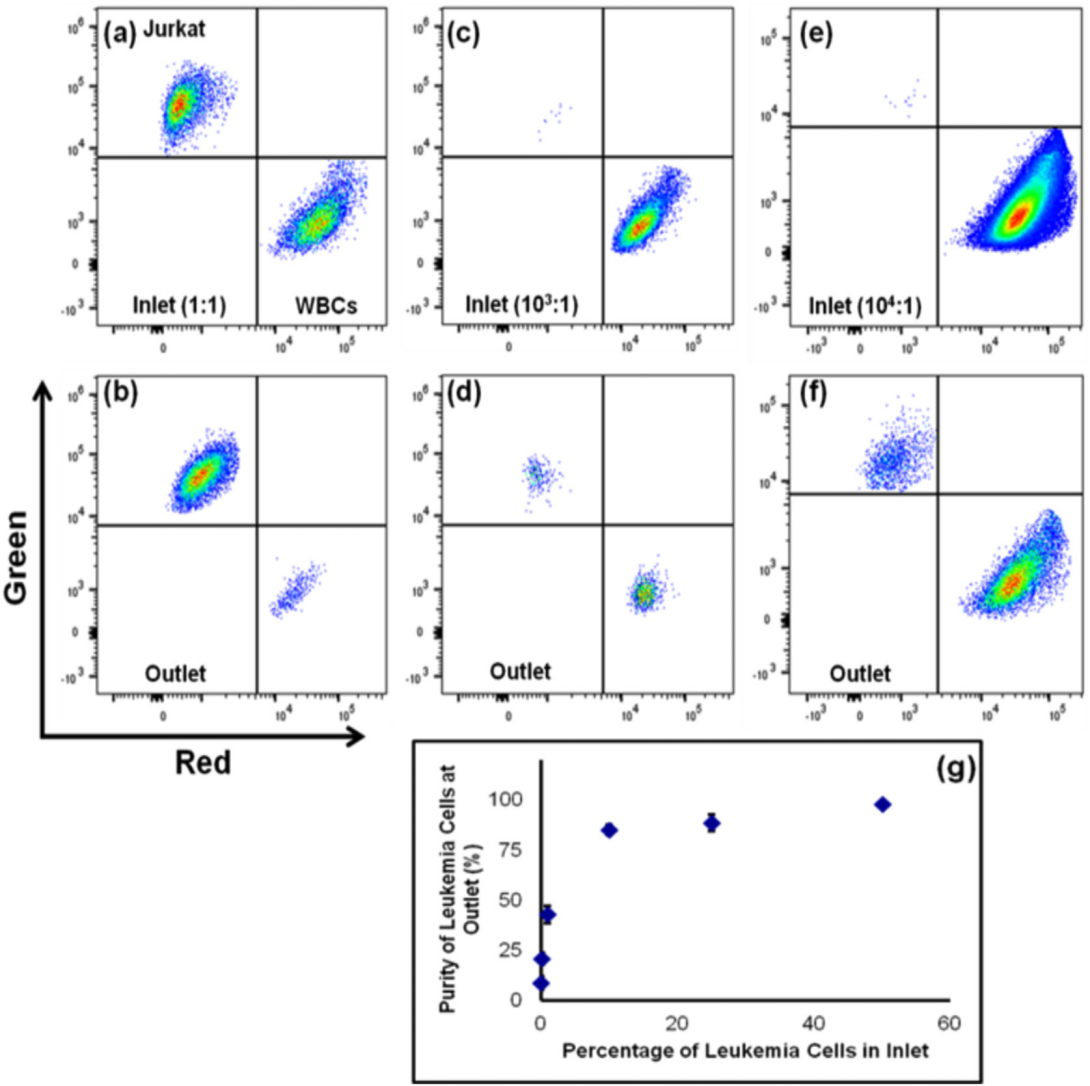
Flow cytometry results showing the data of inlets and outlets for different ratios of cell mixtures (a)–(f). An analysis of Jurkat cell enrichment collected from the soft-1 outlet showing (g) purity, number of independent experiments = 3, number of cells $>10^{4}$, and error bars represent the standard deviation in the experimental data.

To study the accuracy of the sorting, WBCs and Jurkat cells were mixed at various ratios and evaluated at the outlets for purity and enrichment using flow cytometry, with the results shown in Fig. 2. For the equal ratio of 1:1 at the inlet, the purity of leukemia cells at soft 1 outlet was increased to 98.2% with an enrichment factor of ∼8. As the percentage of Jurkat cells at the inlet decreased, the purity of the sorted sample also decreased monotonically although the enrichment factor was higher. In the case of highly dilute Jurkat cells with a ratio of WBC:Jurkat cell of 10^4^:1, the enrichment factor for Jurkat cells was 770. Such high enrichment greatly enhanced the ease of detection and counting of Jurkat cells in flow cytometry analysis, which can be realized from Figs. 2(e) and 2(f). For low percentages of Jurkat samples, a large volume of cell mixture was needed to be processed to detect enough Jurkat cells. In this example of 10^4^ dilution, we processed by flow cytometry a total of 2 million cells in 2 ml of sample, processed at 1000 cells per second, which required over 30 min of processing. After microfluidic isolation, we could reduce the processing time significantly or alternatively improve accuracy of leukemia cell identification.

The overall time needed for flow cytometry processing of 2 million cells was approximately 30 min to count a few events [Fig. 2(e)]. For greater confidence in true positive counts, flow cytometry would require several hours of flow processing. A higher rate of flow processing can be obtained, but in our hands, we suffered in accuracy of counting. Alternatively, the microfluidic enrichment technique processed the 2 million cells in under 30 minutes, but generated a high number of positive counts detectible in flow cytometry in just 10 min of processing [Fig. 2(f)]. For the outlet enriched with Jurkat cells, a relatively large number of cells were detected by analyzing a smaller volume of sample at almost ∼900 times the count rate.

**FIG. 3.**
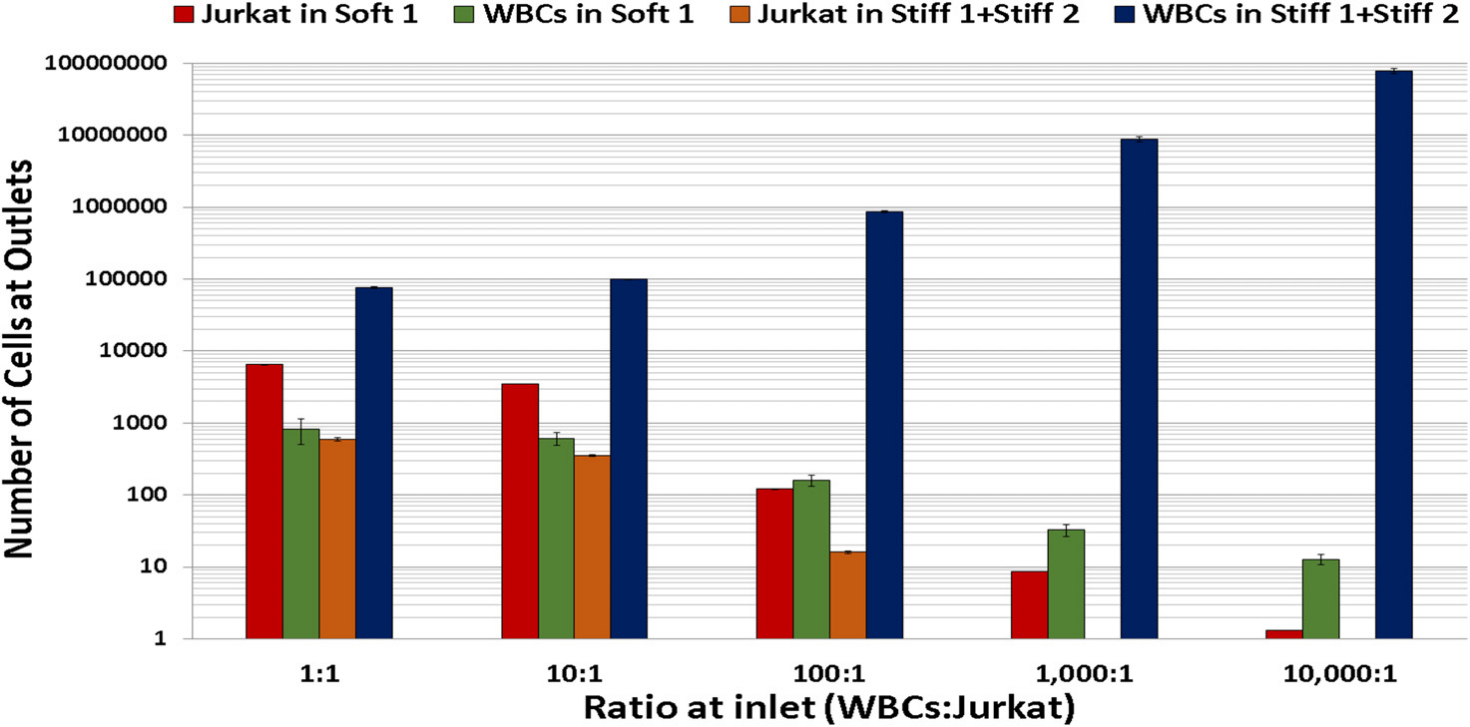
Distribution of WBCs and Jurkat cells in outlets. The soft 1 outlet is enriched with Jurkat cells even for the lower ratio at the inlet. On the other hand, stiff outlets (stiff 1 and stiff 2) are enriched with WBCs (blue) compared to Jurkat cells (yellow), number of independent experiments = 3, number of cells $>10^{4}$, and error bars represent the standard deviation in the experimental data.

To better understand the recovery and collection of target cells, the distribution of WBCs and Jurkat cells was calculated at different outlets and is shown in Fig. 3. For 50% and 10% initial percentages of leukemia cells, the soft-1 outlet collected a majority of Jurkat cells. Even for the low ratio 1:10 000 of initial Jurkat cells to WBCs, the microfluidic device sorted Jurkat cells with very high enrichment by diverting the majority of WBCs to the stiffer outlets although the actual number of WBCs was higher than that of the Jurkat cells in the softer outlets. Also, the number of non-target Jurkat cells collected from the combination of stiffer outlets (stiff 1 and stiff 2) decreased with the lower percentage of leukemia cells in the inlet, indicating a linear response of sorting.

**FIG. 4.**
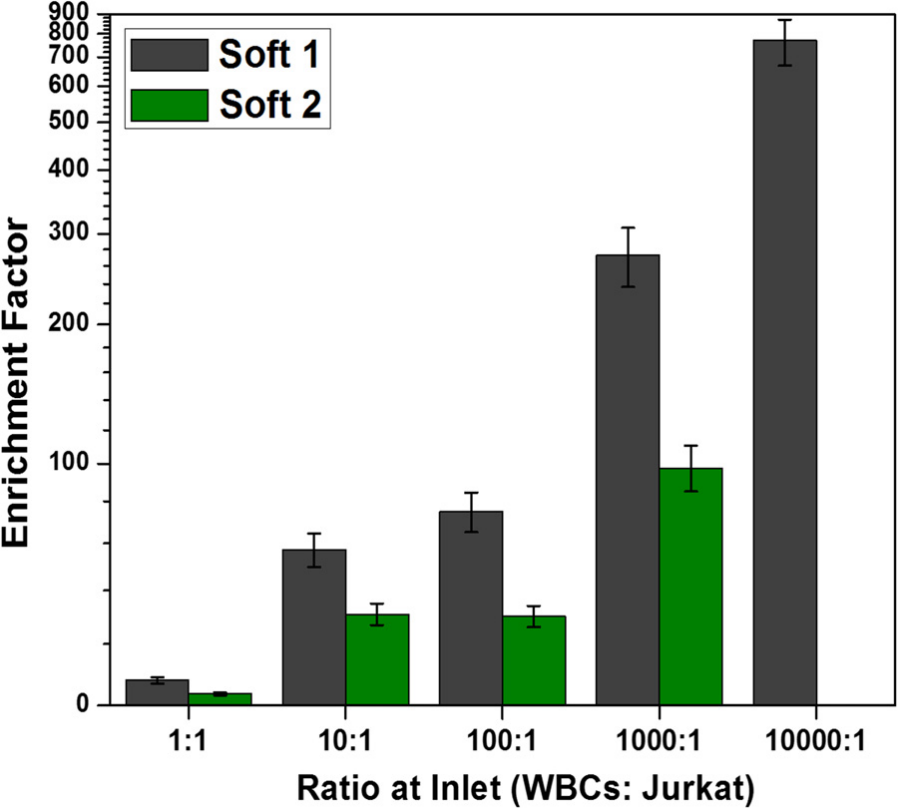
Enrichment factor of Jurkat cells at soft-1 and soft-2 outlets for various WBC:Jurkat cell ratios, number of independent experiments = 3, number of cells $>10^{4}$, and error bars represent the standard deviation in the experimental data.

The enrichment factor of Jurkat cells sorted at soft-1 and soft-2 outlets is shown in Fig. 4. The enrichment factor at a WBC:Jurkat cell ratio of $10^{4}:1$ was determined to be 770 for the soft-1 outlet, which is a significantly higher value. In addition, the enrichment factor at outlet soft-2 ranged from ∼4 at a 1:1 (WBC:Jurkat cell) ratio to ∼100 at a $10^{3}:1$ (WBC:Jurkat cell) ratio. Thus, it is clear that the proposed technique can produce an enrichment factor of cancer cells ranging from ∼4–800. For reference, all the determined enrichment factors are listed in Table S1.

**FIG. 5.**
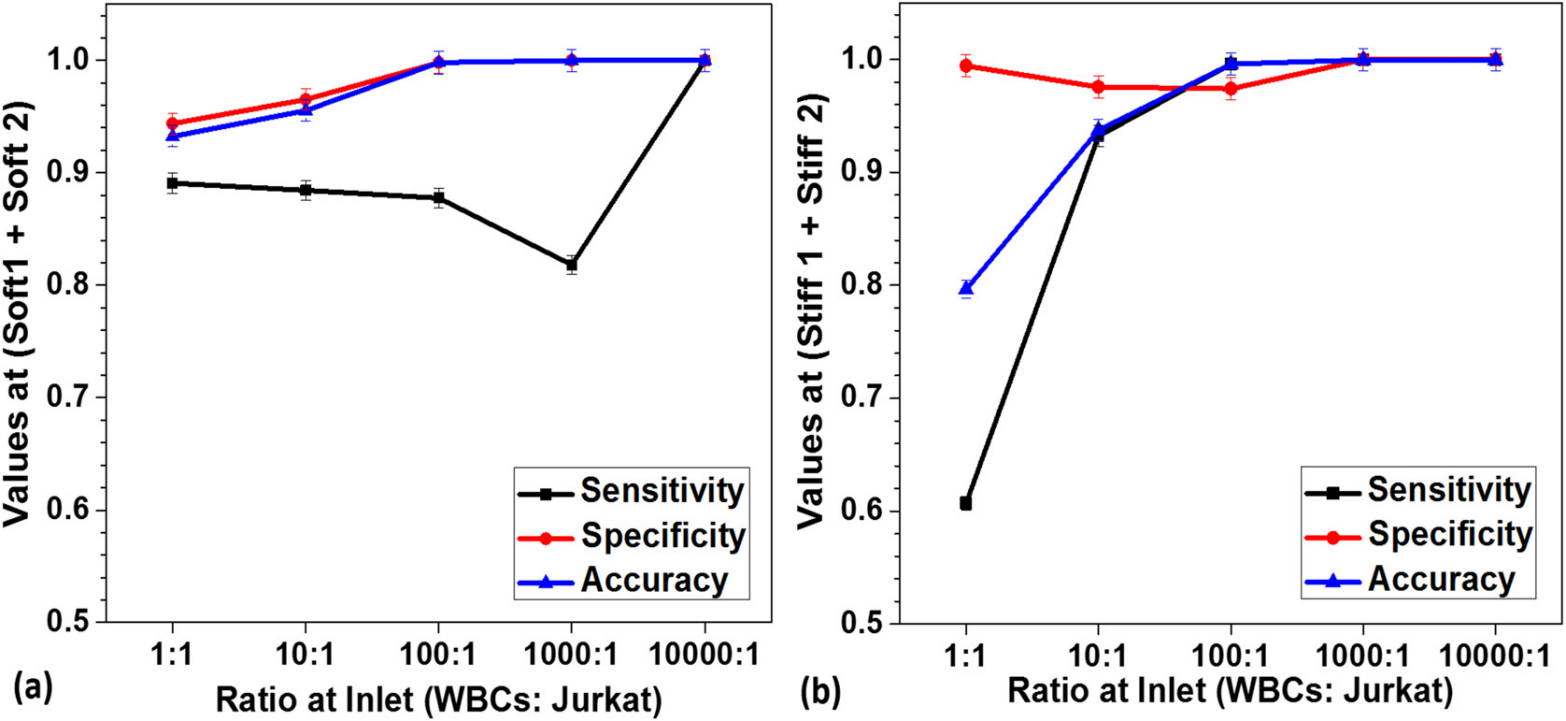
Sensitivity, specificity, and accuracy at (a) (soft-1 + soft-2) outlets and (b) (stiff-1 + stiff-2) outlets at various WBC:Jurkat cell ratios, number of independent experiments = 3, number of cells $>10^{4}$, and error bars represent the standard deviation in the experimental data.

Further, a sensitivity analysis was conducted considering all the five outlets, and the sensitivity, specificity, and accuracy were determined at various WBC:Jurkat cell ratios. Tables S2(a)–S2(c) show how the values were determined in detail. The “middle (soft)” and “middle (stiff)” outlet data were calculated by considering Jurkat cells and WBCs as the true positives, respectively. The sensitivity, specificity, and accuracy for (soft-1 + soft-2) and (stiff-1 + stiff-2) outlets range from ∼0.8−1 for most of the WBC:Jurkat cell ratios, as shown in Figs. 5(a) and 5(b), respectively. For (soft-1 +soft-2) and (stiff-1 + stiff-2) outlets, high sensitivity, specificity, and accuracy show that the sorting method is highly efficient and effective at enriching target leukemia Jurkat cells at even a low Jurkat cell:WBC ratio of 1:10 000. Although the concentration of leukemia cells in the early stage and residual disease can be as low as one cell in 10^6^ WBCs, using our technique, we were able to detect leukemia cells at a concentration of 1 in 10^4^ WBCs efficiently and more quickly.

**FIG. 6.**
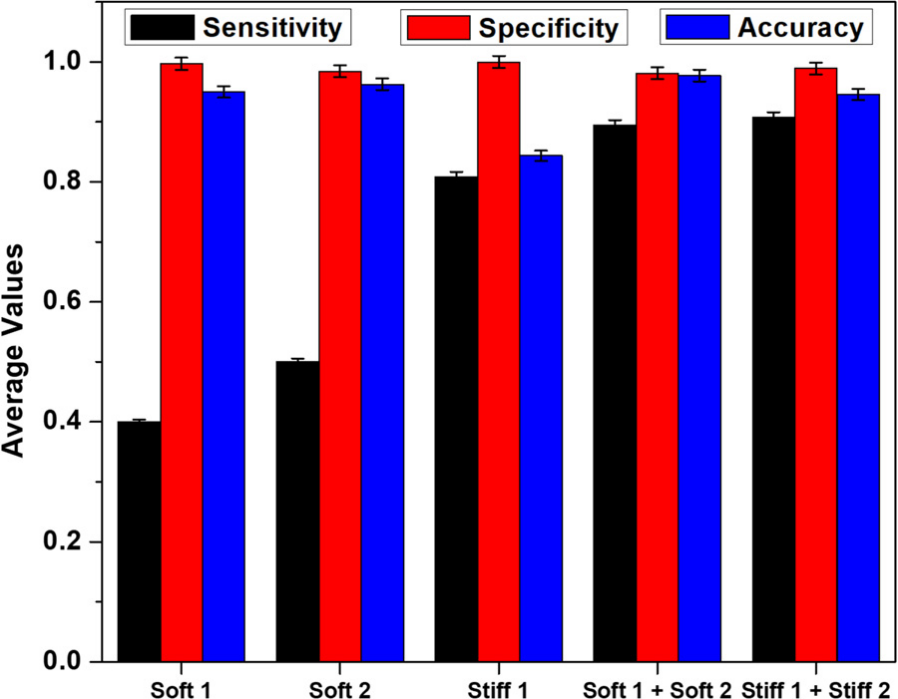
Average values from sensitivity analysis considering various combinations of outlets, number of independent experiments = 3, number of cells $>10^{4}$, and error bars represent the standard deviation in the experimental data.

The average values of sensitivity, specificity, and accuracy (at various WBC:Jurkat cell ratios) were found for all the outlets, which are plotted in Fig. 6. The determined sensitivity, specificity, and accuracy are as high as 0.8–1 for most of the outlet combinations.

### Sorting of leukemia cells from WBCs after chemotherapy treatment

**FIG. 7.**
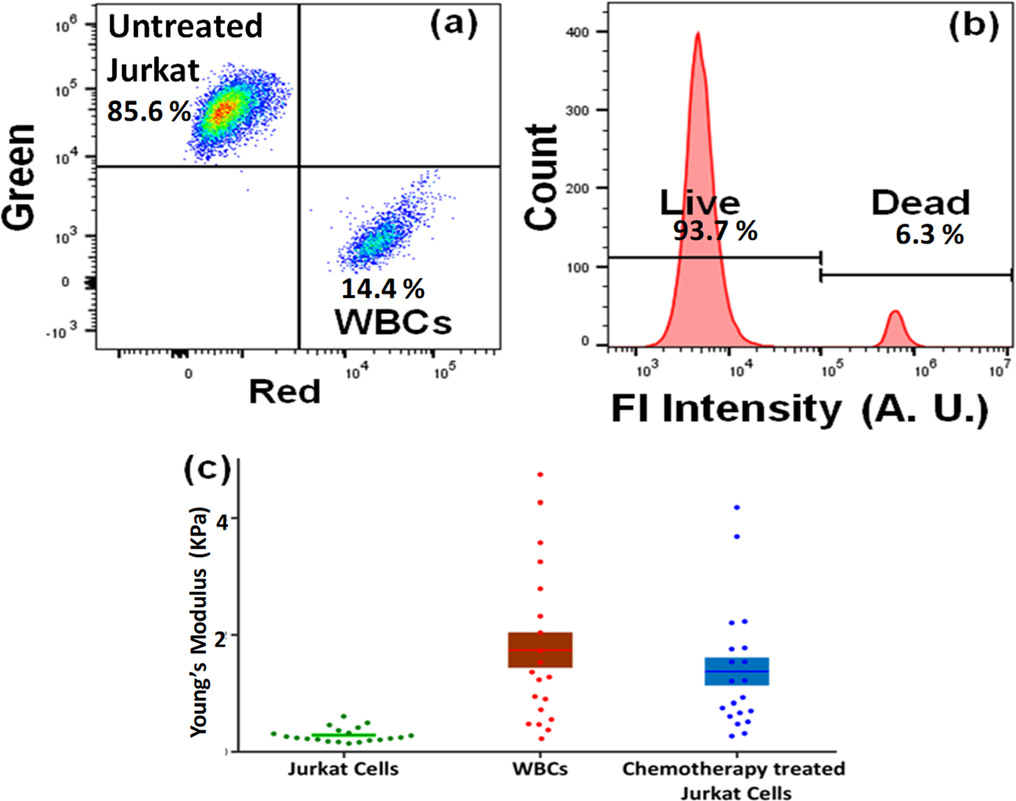
Flow cytometry results to ascertain the (a) percentage of untreated Jurkat cells, Green represents untreated Jurkat cells, Red represents (WBCs + Jurkat cells) treated with daunorubicin, and the data shown represent >100  000 cells; (b) the viability of Jurkat cells and (c) Young's modulus of Jurkat cells, WBCs, and daunorubicin-treated Jurkat cells (p-value < 10^−10^, N = 20–30).

In practical scenarios of analysis of chemoresistance,^33^ complex mixtures of drug-sensitive and drug-resistive cells may be desired to be sorted, to remove the nontarget WBCs and drug-sensitive leukemia cells. Therefore, Jurkat cells and WBCs were mixed at a specified ratio (1:1) and together treated with a standard leukemia chemotherapy drug, daunorubicin. From live/dead stains and flow cytometric analysis, approximately 88% WBCs remain viable after the treatment dose (data not shown). On the other hand, all the Jurkat cells became nonviable due to the treatment. To simulate resistive cells, untreated Jurkat cells were added so that the final percentage of Jurkat cells is 20% untreated Jurkat cells. This mixture, thus, created four classes of cells: drug-treated Jurkat, untreated Jurkat, and both viable and nonviable drug-treated WBCs. The prepared sample was processed by the device with the goal of isolating the untreated Jurkat cells. From the soft-1 outlet, 93.1% viable Jurkat cells were collected and the purity of Jurkat cells was 85.6%, as shown in Figs. 7(a) and 7(b). There was no change in morphology of sorted cells in terms of their shape and size. The untreated Jurkat cells could be collected from chemotherapy-treated Jurkat cells and WBCs because these two cell types were significantly stiffer compared to untreated Jurkat cells as shown in Fig. 7(c). Thus, a label-free approach can potentially be exploited to selectively isolate a desired cell type from multiple classes of cells, including potentially resistive leukemia cells after chemotherapy treatment as the resistive cells remain soft after drug treatment.^33,41^ This concept was also validated using K562 cells through resistivity to daurnorubicin in an earlier study.^33^

An earlier study^33^ revealed that ∼15% of K562 cells showed resistance toward daunorubicin for the specific dose of 50 nM for 15 h, and microfluidic sorting was explored to identify molecular mechanisms of drug resistance to examine heterogeneous responses of cancers to therapies. In this study, K562 cells were also mixed with WBCs at a ratio of 1:1 and the sample was treated with daunorubicin with the same dose and sorted by the microfluidic device. An analysis of the viability of the soft-1 outlet was found to be 92.1% with a purity of 84.3% for K562 cells [shown in Figs. S2(a) and S2(b)]. Therefore, this microfluidic device has potential to sort and study the resistive subpopulation of leukemia cells from samples containing normal WBCs.

As per the definition of “Acute Lymphocytic Leukemia,” if at least 30% of the peripheral blood consists of cancerous lymphocytes, the disease is considered to be Leukemia.^42^ Hence, the WBC:Jurkat cell ratio of 1:1 is in a physiological range. Further, various WBC:Jurkat cell ratios (10:1, 100:1, 1000:1, and 10 000:1) are analyzed for the detection of leukemia cells in the context of minimal levels of disease. These results support that the technique could be helpful for detection of drug responses in Acute Lymphoblastic Leukemia as well as Chronic Lymphocytic Leukemia.

The sorting method utilized in the current work is capable of sorting leukemia cells from WBCs based on biomechanical properties. Two or more cell types with distinct stiffness values can, thus, be sorted. In our previous work, we have reported that ovarian cancer cells with distinct invasive abilities have different stiffness values.^19^ They can also likely be sorted using a device sensitive to stiffness. Similarly, breast cancer cell lines with varying metastatic potential have been reported to have distinct stiffness values.^20,43^ They can also likely be sorted using similar techniques.

## CONCLUSION

This study showed that biophysical sorting can be used to enrich Jurkat leukemia cells from normal white blood cells with high enrichment and accuracy, for the purposes of easier and more accurate detection. We showed that for small numbers of leukemia cells spiked into WBC samples, the leukemia cells can be more quickly counted after label-free microfluidic sorting to eliminate the majority non-target cells from the sample. The unique repeated skew ridge microchannel design is effective in enriching over 760-fold for leukemia cells with a sensitivity, specificity, and accuracy ranging from 0.8 to 1, demonstrated for acute T cell leukemia model Jurkat cells. This sorting approach did not require any labeling of the cells to perform, which offers significant practical advantages over the existing label-based sorting methods. Finally, the potential of the device to isolate resistive subpopulations of leukemia cells to chemotherapy treatment was demonstrated. This label-free enrichment of leukemia cells can potentially improve the quality of downstream analyses such as PCR, flow cytometry, and immunophenotyping.

## METHODS

### Fabrication of microfluidic devices

Microfluidic devices were fabricated in polydimethylsiloxane (PDMS) by replica molding from a SU-8 patterned silicon wafer.^32^ Devices were designed in *AutoCAD*, and flow trajectories were simulated in ANSYS Fluent to ensure no stagnation of flow. The molds were fabricated on a silicon wafer by spin coating SU-8 2007 (SU-8 2007, Microchem Corp.) using a double-layer photolithography process. The molds were characterized by profilometry (Dektak 150 profiler) and optical microscopy, and the ridge and channel heights were measured. Several device parameters influence cell trajectories, which include the ridge gap distance, number of ridges, and angle of ridges. These effects were studied in previous publications,^19–24^ and the ridge angle, total number of ridges, and ridge gap were chosen to be 30°, 14, and 7.5 *μ*m, respectively. The ridge gap of 7.5 *μ*m was chosen to be small enough to compress the cells sufficiently without clogging the device and comparable to an average cell diameter of 15 *μ*m.^22–24^ Five outlet devices were implemented to fractionate the output to improve cell purity.^22–24^ The mold pattern was transferred to polydimethylsiloxane (PDMS) and mixed (10:1, wt/wt) with Sylgard 184 silicone elastomer curing agent (Dow Corning). The PDMS mixture was degassed in a vacuum chamber, poured on the mold, and cured at a temperature of 60 °C for three hours. Inlet and outlet holes were punched using a fresh biopsy punch. The PDMS devices were bonded to glass following an air plasma (PDC-32G Harrick) treatment. Tubing Luer adapters were used to connect the inlets of the device to syringes and to collect cell suspensions from the outlets. To prevent non-specific cell adhesion to the microfluidic channel walls, the device channel was incubated overnight with bovine serum albumin (Sigma Aldrich) at a concentration of 10 mg per ml, at 4 °C.

### Cell preparation

Jurkat (CRL-1990) and K562 (CCL-243) cells were purchased from ATCC. The cells were cultured and maintained in RPMI-1640 medium (Sigma) with the addition of 10% FBS (Atlanta Biologicals). All cells were incubated at 37 °C with 5% CO_2_. Cells were expanded to 80% confluency in cell culture flasks over two days. White blood cells (WBCs) were collected from fresh whole blood collected from deidentified donor. Centrifugation followed by red blood cell lysis buffer (Alfa Aesar) was employed to eliminate red blood cells. The isolated white blood cells were resuspended in PBS. To differentiate cell types in flow cytometry (Accuri C6, BD), WBCs were labeled with 2 *μ*M with CellTracker™ red and Jurkat cells in green (Molecular Probes, Inc.) for approximately 1 h at 37 °C. After labeling the cells with the dye, the accuracy of sorting could be quantified using sensitivity analysis. For studies of the effect of chemotherapy treatment on sorting, all cells were treated with daunorubicin at concentrations of 0.05 *μ*M for 15 h, which was found to induce apoptosis in a vast majority of K562 and Jurkat cells and cause stiffening.^35^ From flow cytometry analysis of the sorted subpopulations, the enrichment factor was calculated using the following equation:

$$\frac{{(\mathrm{Number}\;of\;X\;\mathrm{cells}/\mathrm{Number}\;of\;Y\;\mathrm{cells})}_{\mathrm{Outlet}}}{{\left(\mathrm{Number}\;of\;X\;\mathrm{cells}/\mathrm{Number}\;of\;Y\;\mathrm{cells}\right)}_{\mathrm{Inlet}}}.$$

### Experimental setup of Jurkat enrichment

A mixture of WBCs and Jurkat cells at different ratios (1:1, 10^1^:1, 10^2^:1, 10^3^:1, and 10^4^:1) was generated and suspended in a phosphate buffered saline solution at a concentration of approximately 1 million cells/ml and infused into the microfluidic chip using a syringe pump (PHD 2000, Harvard Apparatus) at specified flow rates. Device flow was formed by three inlet streams including two sheath streams, which provided hydrodynamic focusing of the cells to the central region of the channel.^32^ The cell trajectories were observed by mounting the microfluidic chip on an inverted bright-field microscope (Eclipse Ti, Nikon) and recorded using a high-speed camera (Phantom v7.3, Vision Research) at a frame rate of 2000 frames per second.^22^ The high-speed videos were analyzed using *ImageJ* to obtain cell trajectories. The stiffness of cells from all conditions was measured using atomic force microscopy (AFM, MFP-3D, Asylum Research). To improve cell stability during the AFM measurement, a monolayer of poly-L-lysine (MW 300 kDa, Sigma Aldrich) was applied to gently attach cells to the glass substrate. Silicon nitride cantilevers (spring constant 37.1 pN per nm) with 5 *μ*m beads were used to indent the center of the cells at a rate of 1.5 *μ*m per second. Sufficient force was applied to achieve at least 5 *μ*m deformation, which is in close comparison with microfluidic compression. Cell Young's modulus values were calculated from the force-indentation curves by fit to a Hertzian model to compute an average Young's modulus.^19^ One-way analysis of variance (ANOVA) was performed between Young's modulus of Jurkat cells and WBCs to determine statistical significance.

### Sensitivity, specificity, and accuracy analysis

To evaluate the sorting of Jurkat cells and WBCs, a sensitivity analysis was employed.^36^ The cells from various outlets were classified in the confusion matrix [Fig. S1(a)] as true positive (TP), false positive (FP), true negative (TN), and false negative (FN) corresponding to all the five outlets [Fig. S1(b)]. The number of Jurkat cells was considered as TPs for the soft-1, soft-2, and middle outlets, whereas WBCs were considered to be TPs for the stiff-1 and stiff-2 outlets. The sensitivity was defined as the proportion of true positives correctly sorted by the device, $\mathrm{Sensitivity}=\frac{\mathrm{TP}}{\mathrm{TP}+\mathrm{FN}}$ for each outlet or combination of outlets. A sorting outcome with high sensitivity indicates that a high proportion of the desired cells has been collected at an outlet. In the case of the softer Jurkat cells, we evaluate both the soft-1 and soft-2 outlets, as well as their combination.

Specificity is the proportions of true negatives correctly sorted by the device, $\mathrm{Specificity}=\frac{\mathrm{TN}}{\mathrm{TN}+\mathrm{FP}}$ for each outlet or combination of outlets. A sorting outcome with high specificity indicates that most of the non-desired cells at the corresponding outlets are excluded. For example, stiffer WBCs should be excluded from the soft-1 and soft-2 outlets.

Accuracy is the proportion of true cells (TPs and TNs) evaluated for all populations and outlets. Accuracy indicates the degree of veracity of the sorting outcomes, $\mathrm{Accuracy}=\frac{\mathrm{TN}+\mathrm{TP}}{\mathrm{TN}+\mathrm{TP}+\mathrm{FN}+\mathrm{FP}}.$

## SUPPLEMENTARY MATERIAL

See the supplementary material for the confusion matrix (Fig. S1) and the tables showing detailed values of the enrichment factor (Table S1) and Sensitivity, specificity, and accuracy (Table S2).

## AUTHORS' CONTRIBUTIONS

M.I., A.A., W.L., E.K.W., C.F., and T.S. designed the experiments. H.B. and S.B. performed AFM analysis. M.I. fabricated the devices and performed the microfluidics experiments. M.I., A.A., A.P., G.W., W.L., E.K.W., A.R., and T.S. wrote this manuscript. All the authors have read and approved the final manuscript.
